# Automation of the Timed Up and Go Test Using a Doppler Radar System for Gait and Balance Analysis in Elderly People

**DOI:** 10.1155/2023/2016262

**Published:** 2023-06-29

**Authors:** Racha Soubra, Farah Mourad-Chehade, Aly Chkeir

**Affiliations:** Laboratory of Computer Science and Digital Society (LIST3N), University of Technology of Troyes, Troyes, France

## Abstract

The timed up and go (TUG) test is a simple, valid, and reliable clinical tool that is widely used to assess mobility in elderly people. Several research studies have been conducted to automate the TUG test using wearable sensors or motion-tracking systems. Despite their promising results, the adopted technological systems present inconveniences in terms of acceptability and privacy protection. In this work, we propose to overcome these problems by using a Doppler radar system set into the backrest of a chair in order to automate the TUG test and extract additional information from its phases (i.e., transfer, walk, and turn). We intend to segment its phases and extract spatiotemporal gait parameters automatically. Our methodology is mainly based on a multiresolution analysis of radar signals. We proposed a segmentation technique based on the extraction of limbs oscillations signals through a semisupervised machine learning approach, on the one hand, and the application of the DARC algorithm on the other hand. Once the speed signals of torso and limbs oscillations were detected, we suggested estimating 14 gait parameters. All our approaches were validated by comparing outcomes to those obtained from a reference Vicon system. High correlation coefficients were obtained by comparing the speed signals of the torso (*ρ*=0.8), the speed signals of limbs oscillations (*ρ*=0.91), the initial and final indices of TUG phases (*ρ*=0.95), and the extracted parameters (percentage error < 4.8) obtained after radar signal processing to those obtained from the Vicon system.

## 1. Introduction

According to the “World Population Ageing 2020” report, there were 727 million persons aged 65 years or over in the world in 2020 [1]. This population segment increased from 6% of the total in 1990 to 9% in 2019, and will continue to increase to 16% by 2050 [2]. The number of elderly people is expected to double, reaching over 1.5 billion in thirty years, so one in six people worldwide will be more than 65 years old. Accordingly, as life expectancy continues to rise, several needs and interests of older adults must be considered, such as social protection, health care, and housing need. Plus, an increase in the demand for healthcare services is globally projected [3]. To tackle these problems, many technological innovations have been proposed in the research area and the healthcare market in order to provide a good quality of life for elderly people. One such example is the evaluation of mobility in clinics and/or at home.

A person's ability to change his position or location or move from one place to another by walking and basic ambulation (i.e., mobility) stands out as a key indicator to assess the quality of life in the elderly [4–6].

Gait, balance, and transfer are initially linked to the state of health. They are crucial aspects of independent living and successful aging. However, over 30% of older people experience mobility limitations due to several natural changes that occur with aging (e.g., cognitive impairment, osteoarthritis, muscle weakness, and joint problems) [4]. Generally, mobility limitations lead to undesirable physical, cognitive, and social consequences. They often cause a decline in independence, physical disability, and injuries, and an increase in hospital admissions [6–8]. Faced with this reality, early interventions are required to maintain and regain daily activity levels, ensure healthy aging and achieve a better quality of life over time [4]. The literature reveals that more than thirty-one evaluation tests have been previously proposed to assess gait, transfer, and balance in the elderly [9]. These assessments are often used to identify changes in an individual's mobility, detect early signs of decline, and help in guiding therapeutic interventions [7, 8]. Although these assessments differ from each other regarding their practical characteristics, functional level, and content, most of them are only infrequently performed in clinics and hospitals [9]. In this case, assessment results may not totally reflect the real performance of an elderly person as he tries to walk at his best. Hence, it is remarkable that there is a significant need for continuous gait monitoring daily. Bringing mobility assessment to home may overcome the “one-shot” evaluations by identifying small changes, detecting early-stage problems, and selecting proper intervention once needed.

In line with this, continuous gait monitoring requires wise choices of both the mobility assessment test and technological support. First, an accurate assessment test is recommended to improve the thoroughness of evaluations, determine precise plans of care, and monitor progress better [10, 11]. A lack of agreement on which assessment test to use is present [12]; however, an improper selection can reveal bias in performance results [13]. Second, the technological support presented at home should meet several technological, ethical, economic, and evaluative requirements [14]. For instance, Kleinberger et al. [15] recommended using ambient and unobtrusive systems that can adapt to changing situations or environments and acceptably provide services.

Within this context, we propose the use of a valid, reliable, sensitive to change, and acceptable ambient system that will ensure, without replacing the expertise of healthcare professionals, a continuous mobility evaluation by automating the “timed up and go (TUG)” test at home particularly. This assessment test was chosen based on recommendations from geriatricians and several geriatrics societies, including among others, American, France, British, and Nordic societies [16, 17]. The following section affords details about the TUG test, its practical characteristics and phases (i.e., transfer, walk, and turn), and the interpretation of its results. Then, section III describes the existing technologies for automating this test and our proposed technological system, i.e., the Doppler radar system, as well as the purpose behind this choice.

In this study, we explore using multiresolution analysis techniques and supervised machine learning to automate the TUG test, segment its phases, and extract meaningful gait parameters while performing the test. Furthermore, we aimed to show the validity of our approaches by comparing the obtained results with a reference system. The experimental protocol and our work methodology are detailed in Section 4. Then, our results are shown in Section 5, followed by the discussion and conclusion in Sections 6 and 7, respectively.

## 2. The Timed Up and Go Test

The timed up and go (TUG) test was initially described by Podsiadlo and Richardson in 1991 [18]. The test represents a modified version of the “get up and go (GUG)” test that was originally proposed by Mathias et al. [19] in 1986, and it aims particularly to assess mobility and balance in frail elderly people. Within the TUG test, the geriatrician or caregiver will measure using a stopwatch the time a subject need to get up from an armed chair, walk a distance of 3 meters towards a line marked on the floor, turn around, walk back to the chair, and sit down. Nowadays, the TUG test is widely used to assess motor skills and balance in frail people as well as in people with osteoarthritis [20–22], dementia [23], Parkinson's disease [24], low back pain [25], amputation [26], Alzheimer's disease [27], cerebral palsy [28], stroke [29], and people with risk of falling [30–32]. The test has become one of the most popular and recommended functional assessments due to its several advantages shown hereafter.

Despite its simplicity, the TUG test consists of a series of our basic everyday movements and daily life tasks that could be complex for some people but with high value in assessing gait and balance (i.e., standing up, walking, turning around, and sitting down) [16, 33]. First, the sit-to-stand transfer requires both strength and technique. Second, walking a distance of 3 meters includes an acceleration zone at the start of the walk and another deceleration zone when the person prepares to make a U-turn and when he approaches the chair. In addition, turning around could be difficult, especially for older people who may suffer from balance disorders. Third, the stand-to-sit transfer challenges postural control while adjusting the body position on the chair. Moreover, evaluators can perform TUG evaluations with a few pieces of equipment. They only need a chair with a seat height of 46 cm and an armrest height of approximately 65 cm [18], adhesive tape to mark the turning point, a 3-meter space free of obstruction, and a stopwatch to measure the time taken to complete the test. In addition, previous studies have proved the TUG test's validity, reliability, and responsiveness [18, 34, 35].

Within the test, subjects are required to perform two TUG trials at their usual pace, with their own shoes and technical assistance if needed. One trial is performed to check if all instructions were clear for them, and a second trial is considered as the real TUG test to be timed. The measured time begins with the verbal command “Go” and ends when subjects sit on the chair and rest their back against the backrest of the chair. According to the founder of the test, a total time of 10 seconds or less indicates normal mobility and autonomy in daily life activities [18]. On the other hand, Bohannon [36] carried out a meta-analysis to determine the normative and definitive reference values regarding age. The review included 21 studies with a total of 4395 subjects from North America, Asia, Europe, Australia, and the Middle East. Although some differences could be found while performing the test in the countries mentioned above, however, results revealed that a TUG performance would be below average if time exceeds: 9 seconds in people aged 60 to 69, 10.2 seconds in people aged 70 to 79, and 12.7 seconds in people aged 80 to 99 years.

Generally, the time taken to complete the TUG test is considered a primary outcome for mobility evaluation and fall risk prediction. However, some evaluators examine additional details similar to whether subjects lean forward normally, use the armrests while standing up from the chair, walk straightly, make a turn of 180° without trampling, or make control to sit down. Remarkably, a subject may encounter a specific problem when performing one of the test phases. However, the problem may be masked and unnoticed by measuring the global time required to complete all phases together [37, 38]. Furthermore, a measurement of the global time may not provide enough information to guide healthcare professionals in choosing a suitable intervention. Therefore, timing each phase separately can identify the difficulties that disturb motor activities, so clinicians can propose prevention strategies and guide specific treatments. For example, finding that a subject took too long to get up from a chair but walked well would help the clinician adjust and personalize treatment through physiotherapy, medication, change of diet, and resistance training. Nevertheless, and as declared by Savoie et al. [39], it seems difficult to time each phase through conventional hand-timed methods (ex., stopwatch).

Subsequently, automating the TUG test will ensure continuous supervision and provide an adequate evaluation with complementary parameters of mobility and balance in elderly people. In light of this, several technological supports, briefly described in the following section, have been previously proposed in the literature, each one with its own advantages and disadvantages.

## 3. Technologocial Support for TUG Automation

In recent years, Sprint et al. [40] published a benchmark study on the different technologies proposed in research for TUG automation at home. These technologies are mainly classified into two groups: wearable and nonwearable systems. Wearable systems consist of accelerometers and/or gyroscopes. Their main advantage is that they allow precise and continuous measurement in a multiuser environment by a nonspecialist and the user himself. However, as major drawbacks, users must wear the systems daily, put them correctly on a specific point on the body, charge them regularly, and calibrate them from time to time. These tasks might be considered difficult for elderly people and could embarrass their behavior while walking [40, 41]. On the other hand, nonwearable systems are based on ambient sensors such as cameras and ground-connected sensors (ex., SensFloor, NaviFloor, and so on [42]). Remarkably, this type of technology overcomes the majority of problems encountered by wearable ones. However, they also present some inconvenience. For instance, the camera-based sensors are powered directly from an electrical outlet, and their results are recorded on video media for later medical consultation if necessary. Despite this, these systems face several challenges regarding acceptability as they constitute an invasion of privacy. Hence, the ground connected sensors are hidden; however, they are still expensive and not easily applicable at home.

Accordingly, our novel approach is based on the automation of the TUG test and the segmentation of its phases by using a Doppler radar system set into the backrest of a chair. Recently, new research studies have suggested using such a system in monitoring the daily activities of the elderly, predicting the risk of falls, and calculating a few gait parameters [15, 43]. As an ambient sensor, the Doppler radar system affords several advantages instead of the aforementioned ones. It is a simple to use, low cost, and reliable device that respects the privacy of users and could be implemented in a friendly way.

Basically, a radar system transmits electromagnetic signals and examines the echo received from a distant object to measure its direction, speed, and height. The radar transmitter produces electromagnetic signals that the radar antenna emits in the desired direction. The emitted energy gets reflected by a moving object, and thus the radar receiver performs information extraction from the received signal. Pulsed or continuous waves could be emitted depending on what information is needed (ex., motion detection, localization, and measurement of speed.). Pulse radar systems are often used for long-range detection. However, a continuous wave radar suits our needs better as it is recommended for short and medium range detection. It consists of inexpensive equipment and can measure a target speed based on the Doppler effect.

As its name implies, a continuous wave (CW) radar emits electromagnetic signals continuously. It therefore transmits and receives waves at the same time. When reflected by a moving target, the transmitted signal (at a frequency *f*_*e*_) will be shifted by an amount of *f*_*d*_ via the Doppler effect as follows:

(1) $$\begin{array}{c} \begin{array}{c} f_{d}=\frac{2v_{r}f_{e}}{c} \end{array}\text{,} \end{array}$$

where *c*=3 × 10^8^ m/s is the speed of light and *v*_*r*_ is the target speed.

**Figure 1:**
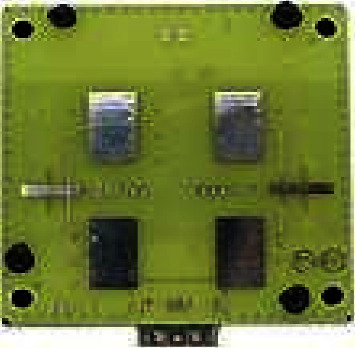
MDU1130 Doppler radar system.

The radar system we used in this study is based on an X-band Doppler Motion Detector that is commercially available by Microwave Solutions LTD [44] and has a carrier frequency (*f*_*e*_) equal to 9.9G Hz, i.e. MDU1130 shown in Figure 1.

## 4. Methodology

### 4.1. Experimental Protocol

The measurement presented in this study was obtained using an MDU1130 Doppler radar system and an optoelectronic Vicon system. This latter was used as a reference system to validate our approaches (i.e., using a radar system in automating the TUG test and segmenting its phases, as well as extracting important gait parameters while performing the test).

Microwave Solutions Ltd provided the Motion Detector Unit (MDU1130). This unit is a miniature microwave Doppler radar optimized for low consumption and low cost. Regarding the radar datasheet, the radiofrequency power levels radiated by the MDU are extremely low under all conditions as the maximum transmitted power is less than 15 mW. This power will be distributed within the coverage pattern of the MDU with a maximum power density of 1 mW/cm^2^ at a distance of 5 mm from the front face of the unit that will also be reduced to 0.72 *μ*W/cm^2^ at a distance of 1 meter. Additionally, it is exempted from the testing requirements for human exposure to electromagnetic fields under the safety aspects of the Radio and Terminal Telecommunication Equipment (R&TTE) directive per EN 62479: 2010, and the emissions are below the maximum permitted exposure levels introduced by the IEEE standard C95.1–1991.

The Vicon system was used with passive markers (VICON System, Oxford Metrics Inc) and consisted of 4 main elements: (1) eight infrared cameras (Bonita 10) with a frame rate of 250 fps, a resolution of 1 megapixel, and a lens operating range up to 13 meters, (2) an acquisition unit (MX-GigaNet) that aims to provide power and data communication to the infrared cameras and other devices, and aims to manage data flow to the Vicon software, (3) an extended MX system with analog capture system (ADC Patch Panel) to incorporate a third-party device (i.e., radar system) with a synchronized acquisition, and (4) a Vicon Nexus software to capture data, manage data acquisition, manage the calibration of the cameras, visualize, and record the results.

Twenty-six healthy subjects, aged between 22 and 60, volunteered to participate in this study, and gave their informed consent. They were asked to perform three TUG trials at slow, normal, and fast speed, leading to a total of 9 trials per subject.

Within the framework of our experiment, the Doppler radar system was placed into the backrest of the chair. It was connected to an electronic circuit in order to amplify its output and filter out noise. The amplification circuit transforms the radar output from millivolts (mV) to volts (V).

The filtration consists of a bandpass filter with two cutoff frequencies of 5 Hz and 500 Hz. The lower frequency is relative to the minimum walking speed (*v*_min_=0.075 m/s); however, the higher frequency is approximately relative to the highest frequency shift that could be generated from the human limbs oscillations. Moreover, the sampling frequency was set to 2500 Hz.

**Figure 2:**
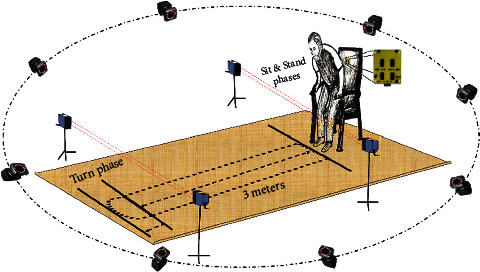
Experimental setup.

On the other hand, eight Bonita 10 cameras were recessed ceiling in order to track the 3D positions of multiple reflective markers placed on the participants' toes, heels, and torso. All cameras were previously calibrated and digitized with a sampling frequency of 250 Hz. Data were synchronized using an analog to digital converter “ADC Patch Panel” provided with the Vicon system, and analyzed on the motion capture software “Nexus 1.8.5” to estimate gait parameters. Our experimental setup is shown in Figure 2.

### 4.2. Doppler Radar Signal Processing

Firstly, the amplified and filtered radar output is a time domain characteristic. This later must be transformed to the spectral domain in order to obtain the frequency shift (*f*_*d*_) information and then calculate the target speed using equation (1) as well as other gait parameters. Accordingly, we propose a signal processing technique based on the continuous wavelet transform (CWT) analysis since it provides a multiresolution analysis while “scaling” and “translating” a predefined wavelet function *ψ*_*a*,*b*_(*t*) over the signal *x*(*t*) as follows:

(2) $$\begin{array}{c} \begin{array}{c} {WT}_{x}^{\psi }\left(a,b\right)=\frac{1}{\sqrt{a}}\int_{-\infty }^{+\infty }x\left(t\right)\psi \left(\frac{t-b}{a}\right)dt\text{,} \end{array} \end{array}$$

where *a* and *b* are the scale and translation parameters, respectively, and $\left(1/\sqrt{a}\right)$ is the energy normalization factor. Additionally, the scale parameter *a* is an alternative notion of the frequency (*f*) with

(3) $$\begin{array}{c} \begin{array}{c} f=\frac{f_{c}}{a} \end{array}\text{,} \end{array}$$

where *f*_*c*_ is the central frequency of the wavelet function *ψ*_*a*,*b*_(*t*). This latter is localized in time and frequency, and is formed based on a predefined mother wavelet *ψ*(∙) as follows:

(4) $$\begin{array}{c} \begin{array}{c} \psi_{a,b}\left(t\right)=\frac{1}{\sqrt{a}}\psi \left(\frac{t-b}{a}\right) \end{array}\text{.} \end{array}$$

The wavelet function *ψ*_*a*,*b*_(*t*) is compressed when *a* < 1 and dilated when *a* > 1. Accordingly, the entire time and frequency axis will be scanned by varying the parameter *b* (i.e., time parameter) and by compressing and/or dilating the parameter *a*, respectively. Hence, a high time resolution and low frequency resolution will be obtained in the analysis of high frequencies; however, a high frequency resolution and low time resolution will be obtained in the analysis of low frequencies [45].

The coefficient *WT*_*x*_^*ψ*^(*a*, *b*) is defined as the dot product of the signal *x*(*t*) and the wavelet function *ψ*_*a*,*b*_(*t*) and represents the correlation between them. When *x*(*t*) oscillates at the same frequency as the wavelet, their dot product becomes maximum; thus the value *WT*_*x*_^*ψ*^(*a*, *b*) is high. On the other hand, if they oscillate with different frequencies, *WT*_*x*_^*ψ*^(*a*, *b*) becomes close to zero. Therefore, this coefficient is proportional to the signal's energy and measures its oscillations at the scale *a* and around the time *b*. The squared modulus of *WT*_*x*_^*ψ*^(*a*, *b*) gives a 2D representation of the signal through a wavelet spectrogram known as “scalogram.” This later displays the energy distribution of the signal.

**Figure 3:**
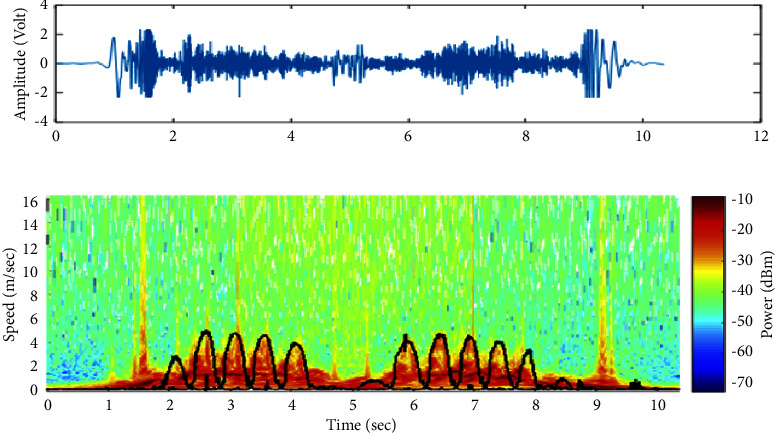
An example of (a) a radar signal registered while performing the TUG test and (b) its scalogram. Signals in black represent the speed signals of the lower limb oscillations (i.e., toes) obtained from the Vicon system.

In this study, we have analyzed a large number of our amplified and filtered radar signals by the “Bump” wavelet transform based on previous results [46]. An example of an output signal and its scalogram are shown in Figures 3(a) and 3(b), respectively. As seen in Figure 3(a), the signal's amplitude decreases when subjects walk away from the Doppler radar system. On the other hand, the scalogram (Figure 3(b)) shows the frequency components of the signal in function of time, where the *x*-axis represents the time in seconds and the *y*-axis represents the Doppler frequency shift; hence, the speed of the human body, including the lower and upper limbs oscillations, is in (meter/seconds) (noting that the speed is proportional to the frequency and inversely proportional to the wavelet scale parameter based on equations (1) and (3), respectively). Each point of the scalogram is color-coded according to the signal's energy in de cibels(dB m); where red color refers to the highest intensity and blue color refers to the lowest ones. The signal in black represents the reference speed signals of the lower limbs oscillations (i.e., right and left toes) obtained from the Vicon system. It is noticeable that the maximum energy distribution corresponds to the speed signal of the torso as it occupies the largest surface across the radar system. However, the maximum envelope in the scalogram corresponds to the speed signals of right and left toes as they produce the highest oscillations.

Let **M**(*a*, *b*) be the squared modulus of *WT*_*x*_^*ψ*^(*a*, *b*). Thus, the maximum energy *mx*(*t*) could be extracted from the matrix coefficient **M** at each instant as follows:

(5) $$\begin{array}{c} mx\left(t\right)=v_{torso}\left(t\right)\\ =\max_{a}M\left(a,b\right)\text{.} \end{array}$$

**Figure 4:**
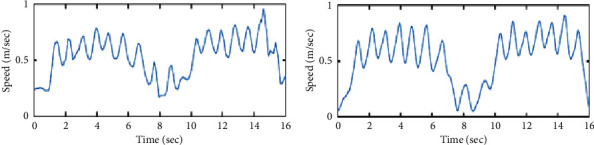
The torso's speed signal is obtained from (a) the Doppler radar system and (b) the reference Vicon system.

The Pearson correlation coefficients between *mx*(*t*) signals and the resampled ones obtained from the Vicon system (i.e., the reference speed signals of the torso) were calculated in order to validate the use of our Doppler radar system in automating the TUG test. An example of both speed signals of the torso (radar vs. Vicon) is illustrated in Figures 4(a) and 4(b), respectively. Furthermore, in order to extract the speed signal of toes oscillations (i.e., the maximum envelope), we notice, with reference to the Vicon results (i.e., signal in black in Figure 3(b)), that the energy distribution values presented under and above this signal differ from each other. Accordingly, we propose a classification technique between the energy values to automatically detect the maximum envelope and thus automatically segment the TUG phases and extract further gait parameters.

Let *v*(*t*) be the reference speed signal of toes oscillations, so in other words, the derivative of the markers' displacement which are placed on the right and left toes with respect to the *x*-axis is as follows:

(6) $$\begin{array}{c} \begin{array}{c} v\left(t\right)=\frac{\partial x_{RTOE}}{\partial t}+\frac{\partial x_{LTOE}}{\partial t} \end{array}\text{.} \end{array}$$

As shown in the scalogram of Figure 3(b), the energy distribution values that are under *v*(*t*) refer to the upper and lower limbs oscillations; however, those that are present above *v*(*t*) refer to the environmental noise. Accordingly, we propose a classification technique that is able to separate between these two groups of energy based on K-means approach.

The K-means approach refers to unsupervised learning that is most commonly used for clustering purposes [47–50]. It partitions a set of data into **k** homogeneous groups, known as clusters and where **k** represented the predefined number of groups, such that the within-cluster variance is minimized as much as possible (i.e., high intraclass similarity).

Assume *x*_*i*_*ϵℝ*^*p*^ is the set of points presented in the matrix **M** where *iϵ*{1 … *n*}, and to be classified into 2 groups or clusters (**k**=2). The idea behind the K-means approach is to assign an arbitrary centroid *c*_*j*_ for each cluster *C*_*j*_, where *j* *ϵ*{1,…, **k**}, and then set each point *x*_*i*_ to their closest *c*_*j*_ by measure of similarity between them (i.e., measurement of the Euclidian distance).

Afterward, the algorithm computes the new mean values of each cluster to be assigned as new centroids. Now that the centroids are recalculated, every point *x*_*i*_ is reassigned if it became closer to another cluster. The centroid recalculation and cluster assignments steps are iteratively repeated until we achieve convergence in clustering or until a predefined number of iterations.

The K-means algorithm is summarized as follows:

1. Select the number of clusters **k**
2. Select the initial centroids randomly *c*_1,_*c*_2_,…, *c*_*k*_
3. Calculate the distance *d*_*i*_ between each data point *x*_*i*_ and the centroids
4. *x*_*i*_ ∈ *C*_*j*_⟷*d*=arg min ‖*x*_*i*_ − *c*_*j*_‖^2^
5. Assign each data point to the nearest cluster *C*_*j*_ with respect to *d*_*i*_
6. *C*_*j*_=set of closest points to *c*_*j*_
7. Recalculate the new centroid for each cluster *C*_*j*_
8. *c*_*j*_=(1/*N*_*j*_)∑_*x*_*i*_∈*C*_*j*__*x*_*N*_
9. where *N* is the number of data points in the cluster *C*_*j*_
10. Repeat steps 3 to 5 until convergence in clusters or until a predefined number of iterations.

The simplicity of the K-means algorithm made its use in various fields. However, clustering through this approach is very sensitive concerning the initial centroids. Yet, they are firstly selected arbitrarily. In some cases, incorrect results may be obtained if the initial centroids are far from the final cluster centre [51]. Accordingly, we propose a semisupervised learning strategy in which the initialization of the centroids will be automatically selected based on a preclassification of our data point into 2 groups: “oscillations cluster” and “noise cluster.” In this approach, we aim to detect the threshold value (**th**) that separates the two groups of energy based on a baseline signal (i.e., radar data collected in the absence of a moving target).

**Figure 5:**
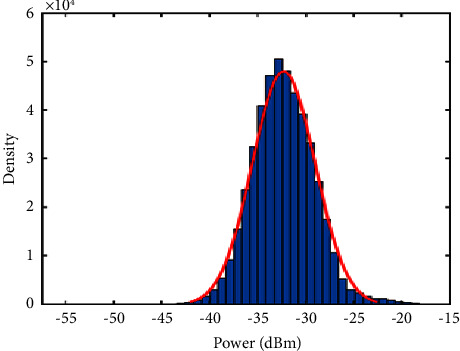
Histogram of the energy distribution obtained from the radar system in the absence of a moving target.

The histogram in Figure 5 shows the data distribution related to noise and their normality. This later has also been verified by applying the Kolmogorov–Smirnov test [52]. On that basis, 95% of these data vary between *μ* − 1.96*σ* and *μ*+1.96*σ*, where *μ* and *σ* refer to the mean and standard deviation of the data, respectively. Thus, we can estimate that **th** is equal to *μ*+1.96*σ*, as the energy values related to the limbs oscillations are superior to those related to noise (Figure 3).

**Figure 6:**
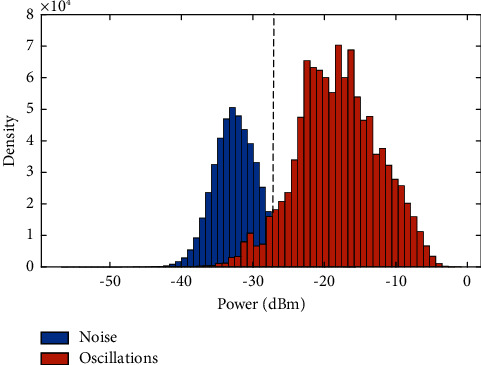
Histogram of the energy distribution values related to limb oscillations and noise in comparison to *th* value.

Figure 6 shows an example of the energy distribution values that are related to noise (in blue) and those related to limb oscillations (in orange) with reference to the signal *v*(*t*) and in comparison with **th** value. Almost all data are correctly grouped, however, some elements that belong to limbs oscillations and are present under the signal *v*(*t*) will be eliminated after applying **th** as they are very close to the noise values. Therefore, we proposed to assign proper values to these elements based on morphological processing [53, 54]. This mathematical methodology compares the coefficient matrix to be analysed by a set of known geometry called a structuring element. The basic morphological operations are erosion and dilation, and their combination allows the production of other processes, such as closing (dilation followed by erosion) and opening (erosion followed by dilation).

In this work, we applied the closing operation on the matrix **M** in order to plug the unwanted holes (i.e., noise values in *v*_inf_), connect the disjoint points and keep the contours. Three main properties of the structuring element must be considered: shape, size, and origin [55]. The optimal structuring element B was selected based on the results obtained compared to those obtained from the reference Vicon system.

The closure operation is a combinational operation of dilation followed by erosion, and is defined as follows:

(7) $$\begin{array}{c} \begin{array}{c} M.B=\left(M⊕B\right)⊝B\text{,} \end{array} \end{array}$$

where ⊕ represents the sign of dilation and ⊝ represents the sign of erosion, with

(8) $$\begin{array}{c} A=M⊕B\\ =\left\{z\left|{\left(B\right)}_{z}\cap M\neq \emptyset \right.\right\}\text{,}\\ A⊝B=\left\{z\left|{\left(B\right)}_{z}\subseteq A\right.\right\}\text{.} \end{array}$$

Consequently, we set the initial centroids to the mean values of each group and apply the K-means' steps 3 to 5 until convergence in clusters. The matrix **M** will be transformed into a dichotomous matrix **M**′; thus, we identify the speed signal of toes oscillations *v*_toe_(*t*) by detecting the maximum envelope separating between the values of one and two in each column of **M**′.

The correlation coefficients between *v*_toe_(*t*) signals and the reference ones obtained from the Vicon system *v*(*t*) were calculated in order to validate our methodology.

**Figure 7:**
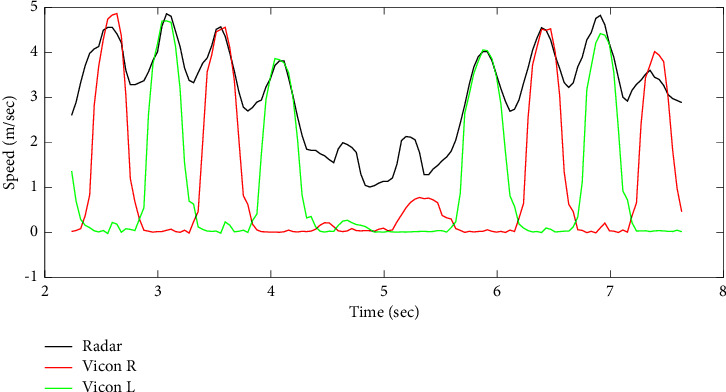
Speed signal of toes oscillations obtained from the Doppler radar system (in black) and the reference Vicon system (in red and green for right and left toes, respectively).

An example of both speed signals of toes oscillations (radar vs. Vicon) is illustrated in Figure 7.

### 4.3. Automatic Phase Segmentation

As mentioned, the TUG phases are three: (1) transfer (including standing up and sitting down components), (2) walk, and (3) turn. This section suggests a TUG test segmentation based on each phase's starting and ending points. Thus, two segmentation approaches were proposed: one for “transfer-walk segmentation” and a second for “walk-turn segmentation.”

#### 4.3.1. “Transfer-Walk” Segmentation

The purpose behind the segmentation between “transfer” and “walk” phases is to separate each of the components of standing up and sitting down from the walking phase in a TUG test. Our proposed method is based on the detection of the first and the last walking steps. The first walking step indicates the ending point of getting up from the chair as well as the starting point of walking. On the other hand, the last walking step indicates the ending point of walking phase and the starting point of sitting down.

**Figure 8:**
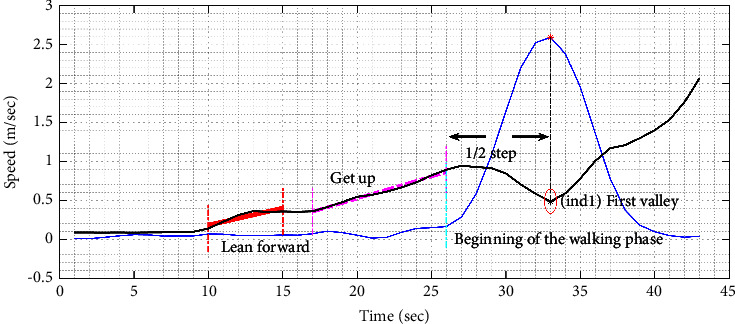
Speed signal of the torso obtained from the radar system (in black) and speed signal of toes oscillations obtained from the Vicon system (in blue).

The speed signals of torso *v*_torso_(*t*) and toes oscillations *v*_toe_(*t*) have been extracted after processing the radar signals. In comparison with the reference signals obtained from the Vicon system, it is clear that the peak values in *v*(*t*) (i.e., *v*_toe_(*t*)) highly correlate with the bottom points in *v*_torso_(*t*) (Figure 8). Let *I*_osc_ be the time index that represents the first oscillation phase of the walking cycle and segments between transfer and walk phases as follows:

(9) $$\begin{array}{c} I_{osc}=I_{val}-hsl, \end{array}$$

where *I*_val_ represents the time index of the first bottom point in *v*_torso_(*t*) and *hsl* indicates the length of a half step (half step length, equations (25) and (26)). The correlation coefficient between the estimated and the real time indices calculated as follows:

(10) $$\begin{array}{c} r=\frac{Cov\left(v_{E},v_{R}\right)}{\sigma_{v_{E}}\sigma_{v_{R}}}\text{,} \end{array}$$

where Cov(*v*_*E*_, *v*_*R*_) is the covariance between estimated (*v*_*E*_) and real time (*v*_*R*_) vectors, and *σ*_*v*_*E*__ and *σ*_*v*_*R*__ is the standard deviations of these vectors, respectively.

#### 4.3.2. “Walk-Turn” Segmentation

In this section, we propose a segmentation between “walk” and “turn” phases based on the automatic detection of the first and the last time indices of turning by applying the DARC algorithm [56]. First, different signals were obtained by extracting statistical parameters from the columns of the squared modulus of *WT*_*x*_^*ψ*^(*a*, *b*) (i.e., **M**(*a*, *b*) matrix), such as mean, standard deviation, variance, and kurtosis. [57]. For example, the variance was calculated as follows:

(11) $$\begin{array}{c} v\left(b\right)=\underset{a}{var}M\left(a,b\right)\\ \begin{array}{c} =\frac{\sum_{a}{\left(M\left(a,b\right)-E\left(M\left(a,b\right)\right)\right)}^{2}}{N}, \end{array} \end{array}$$

where *E*(**M**(*a*, *b*))=(∑_*a*_**M**(*a*, *b*)/*N*) represents the mean of coefficients for a fixed value of *b,* and *N* represents the number of elements in *a*.

Afterward, the DARC algorithm was applied on the extracted signals by following the steps:

(1) Normalize *v*(*b*) data by dividing each value by the maximum value of the signal in order to obtain *v*_1_(*b*) signal in which values vary between 0 and 1
(2) Apply a variance filter to *v*_1_(*b*)
(3) Calculate the cumulative values of the signal
(4) Search for a scanning window:
  - Multiply each value of the cumulated signal by a factor of 10^4^ and round it to the nearest integer
  - Slide, in steps of two, a “window” of 200 points
  - Determine the number of points in each window: if the number of points found corresponds to a time period greater than 0.5 sec, the processor increments a “window” counter and retains the time index of the first and the last point of the window
  - Transform signal into binary by replacing the values located between the time indices of the “window” into 1, and outer values into 0.

Finally, the percentage of error between the ground truth values and our estimated values for the initial and end points of turning phase has been calculated as follows:

(12) $$\begin{array}{c} \begin{array}{c} \%\;error=\frac{V_{DARC}-V_{Vicon}}{N}\times 100 \end{array}\text{,} \end{array}$$

where *V*_DARC_ and *V*_Vicon_ represent the output index obtained from the DARC algorithm and Vicon system, respectively, and *N* is the length of turn phase.

### 4.4. Derived Gait Parameters

With age, several gait parameters change under the effect of physiological and/or pathological aging [58].

These changes are mainly characterized by a decrease in walking speed, an irregularity in the steps, a decrease in the arms swing, and an increase in double support time [59–62]. However, an abnormal variability of these parameters can be considered as a pathology for an elderly person. Some authors consider this variability to be a predictor of the risk of falls, loss of autonomy, institutionalization, and death [62–64]. In particular, spatiotemporal parameters are valid descriptors of walking [61]. Their measurement may concretize a subject's performance and characterize its overall walk.

**Figure 9:**
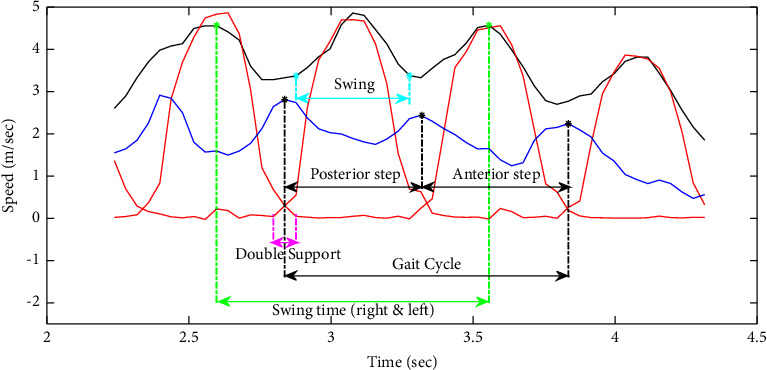
Example of the speed signals of the torso (in blue) and toes oscillations obtained from the radar system (in red) and the Vicon system (in black) while walking.

As shown in Figure 9, peaks and bottom points of *v*_torso_(*t*) and peaks of *v*_toe_(*t*) are key indicators for the evaluation of spatiotemporal parameters.

Let {*j*_1_, *j*_2_,…, *j*_*N*_}, {*i*_1_, *i*_2_,…, *i*_*N*_}, and {*i*_2_, *i*_3_,…, *i*_*N*+1_} be the peaks of *v*_torso_(*t*), the bottom points of *v*_torso_(*t*), and the peaks of *v*_toe_(*t*), respectively. Accordingly, the following equations were proposed in order to calculate:

(a) Number of steps:where a step corresponds to a single right or left step
  (13) $$\begin{array}{c} Nb\;of\;steps=N\text{.} \end{array}$$
(b) Step time (sec): time elapsed from initial contact of one foot to initial contact of the opposite foot
  (i) Anterior step:
    (14) $$\begin{array}{c} \begin{array}{c} {Step\;Time}_{ant}=j_{k+1}-j_{k}\text{.} \end{array} \end{array}$$
  With *k* ∈ {2,4,…, *N* − 2} if *N* is even and *k* ∈ {2,4,…, *N* − 3} if *N* is odd.
  (ii) Posterior step:
    (15) $$\begin{array}{c} \begin{array}{c} {Step\;Time}_{post}=j_{k+1}-j_{k}\text{.} \end{array} \end{array}$$
  With *k* ∈ {3, 5,…, *N* − 3} if *N* is even and *k* ∈ {3, 5,…, *N* − 2} if *N* is odd.
(c) Gait cycle duration (sec): interval of time between repetitive events of walking
  (16) $$\begin{array}{c} \begin{array}{c} Gait\;Cycle=j_{k+2}-j_{k}\text{,} \end{array} \end{array}$$
with *k* ∈ {2,3,…, *N* − 2}.
(d) Swing time (sec): time elapsed between the last contact of one foot (toe off) and the initial contact of the same foot (heel contact)
  (17) $$\begin{array}{c} \begin{array}{c} Swing\;Time=i_{k+2}-i_{k}\text{,} \end{array} \end{array}$$
with *k* ∈ {2,3,…, *N* − 2}.
(e) Average walking speed (cm/sec):
  (i) Walk#1
    (18) $$\begin{array}{c} \begin{array}{c} {speed}_{walk\#1}=\frac{\overset{t=t_{2}}{\underset{t=t_{1}}{\sum }}v_{torso}\left(t\right)}{t_{2}-t_{1}}\text{.} \end{array} \end{array}$$
  *t*_1_=*j*_1_ (i.e. start of a walking phase) and *t*_2_ represents the time index of the end of the first walking phase detected by the DARC algorithm.
  (ii) Walk#2
    (19) $$\begin{array}{c} \begin{array}{c} {speed}_{walk\#2}=\frac{\overset{t=t_{4}}{\underset{t=t_{3}}{\sum }}v_{torso}\left(t\right)}{t_{4}-t_{3}}\text{.} \end{array} \end{array}$$
  *t*_3_ represents the time index of the start of the second walking phase detected by the DARC algorithm and *t*_4_=*j*_*N*_.
(f) Cadence (steps/min): number of steps per minute
  (20) $$\begin{array}{c} Cadence=\frac{1}{Gait\;Cycle}\times 60\\ \begin{array}{c} =\frac{60}{j_{k+2}-j_{k}} \end{array}\text{,} \end{array}$$
with *k* ∈ {2,3,…, *N* − 2}.
(g) TUG walking phase duration (sec): time is taken to walk 3 meters
  (21) $$\begin{array}{c} \begin{array}{c} {Time}_{walk}=j_{N}-j_{1}\text{.} \end{array} \end{array}$$
(h) TUG duration (sec): time is taken to perform all TUG phases
  (22) $$\begin{array}{c} \begin{array}{c} {Time}_{TUG}=\sum {Time}_{transfer+walk+turn}\text{.} \end{array} \end{array}$$
(i) Step length (cm): anterior-posterior distance measured from the heel contact of one foot to the heel contact of the opposite foot
  (i) Anterior step
    (23) $$\begin{array}{c} \begin{array}{c} {Step\;L}_{ant}=\frac{{Step\;Time}_{ant}\times 3}{{Time}_{walk}} \end{array}\text{.} \end{array}$$
  (ii) Posterior step
    (24) $$\begin{array}{c} \begin{array}{c} {Step\;L}_{post}=\frac{{Step\;Time}_{post}\times 3}{{Time}_{walk}} \end{array}\text{.} \end{array}$$
(j) Stride length (cm): anterior-posterior distance measured between two consecutive heel contacts of the same foot (e.g., left-to-left or right-to-right); also defined by two steps (ex., a right step followed by a left step)
  (i) Anterior step
    (25) $$\begin{array}{c} \begin{array}{c} {Stride\;L}_{ant}={Step\;L}_{ant}+{Step\;L}_{post}\text{.} \end{array} \end{array}$$
  (ii) Posterior step
    (26) $$\begin{array}{c} \begin{array}{c} {Stride\;L}_{post}={Step\;L}_{post}+{Step\;L}_{ant}\text{.} \end{array} \end{array}$$

**Figure 10:**
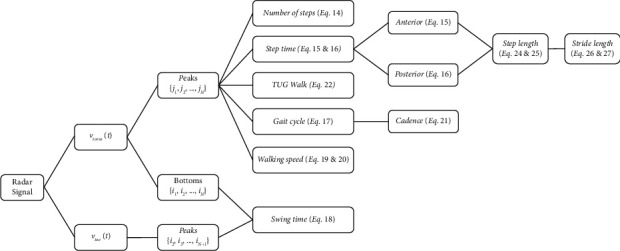
Flowchart of the derived gait parameters.

The flowchart in Figure 10 summarizes the steps in the derivation of gait parameters.

## 5. Results

All our approaches were validated in this work through an optoelectronic Vicon system installed in the experimental area. The radar and Vicon data acquisition were synchronized using an analog device. Vicon data were resampled to the same number of radar data points to compare them.

### 5.1. Validity of Speed Signals

A comparison of gait speed signals (i.e., speed signals of the torso) obtained from the wavelet scalogram and those obtained after 3D localization of the torso marker attached to the participant was performed. An example of this comparison is shown in Figure 4, and a correlation coefficient equal to 0.8 was obtained.

In order to detect speed signals of toes oscillations, we proposed a semisupervised machine learning. The baseline signal revealed a threshold value (**th**) equal to −27  de cibels. As shown in the histogram of Figure 6, clustering the energy distribution values that are related to noise and those related to limbs oscillations by (**th**) was encouraging.

However, an improvement has been made by applying the morphological operation of closing with a structuring element **B** of rectangular shape, size (3 × 9), and centered origin. The energy values related to the oscillations, and having a value close to those of the noise, have been converted into correct values (similarly for the noise values). The comparison between the speed signals of limbs obtained by the Doppler radar system and those obtained by the Vicon reference system demonstrated a higher correlation after applying the morphological operation on the matrices, with *ρ*=0.87 and *ρ*=0.91 before and after the closing process.

**Figure 11:**
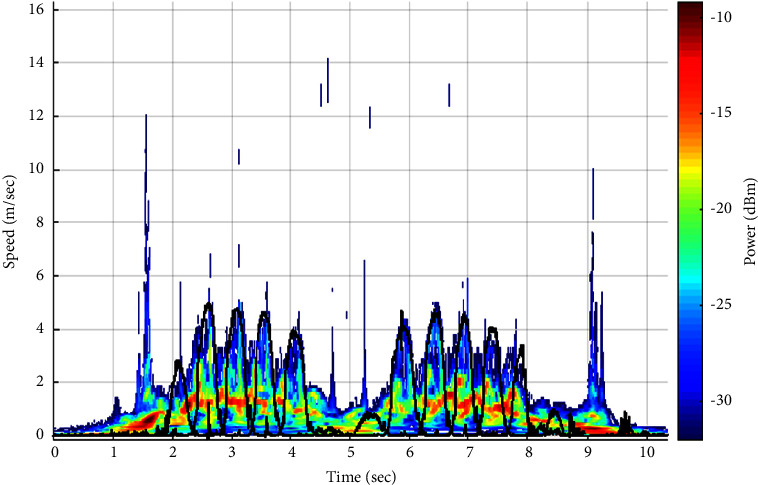
New partition of the matrix *M* after the application of the semisupervised K-means algorithm.

Figure 11 shows an example of partitioning the matrix data into two parts. Outcomes revealed a number of iterations between 12 and 14 for the convergence of the K-means algorithm, and a correlation coefficient between speed signals equal to 0.89. Both correlation coefficients prove the validity of our proposed approaches in automating the TUG at home and calculating gait speed through a Doppler radar system.

### 5.2. TUG Phase Segmentation

**Figure 12:**
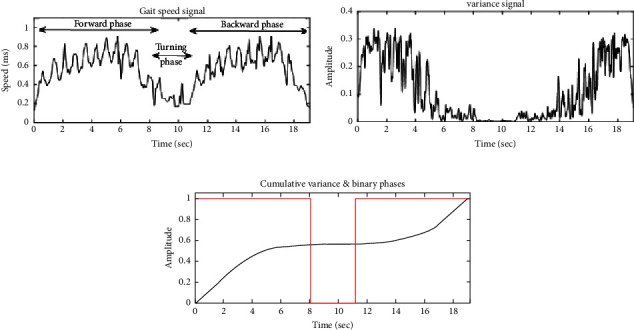
An example of (a) an extracted gait speed signal and (b) the variance signal extracted from the wavelet coefficient matrix with (c) its cumulative data signal and the automatic detection of the turning phase.

Two approaches were proposed and tested on a maximum number of trials to segment the TUG phase. For “transfer-walk segmentation,” our outcomes revealed correlation coefficients between time indices greater than 0.95 and mean squared error values equal to 0.09 seconds. For “walk-turn segmentation,” ten statistical parameters were extracted from the matrix, leading to ten reconstructed signals. Applying the DARC algorithm on the variance signal ensured the lowest possible error rate equal to 0.13 seconds.  Figure 12(a) shows an example of an extracted gait speed signal, (b) the variance signal extracted from the wavelet coefficient matrix with (c) its cumulative data signal, and the automatic detection of the turning phase [65].

### 5.3. Prediction of Gait Parameters

Using the speed signals of torso and limbs oscillations, we extracted 14 gait parameters.  Table 1 reports the error rates of the estimation of parameters (following equation (12)). The error is low and varies between 0 and 4.8%.

## 6. Discussion

The main purpose of this work was to automate an accurate mobility assessment test in order to provide a continuous evaluation in an objective and user-friendly way for the elderly. Currently, healthcare professionals refer to the TUG test time and subjectively analyze a subjects' performance in this test to identify early mobility decline, assess mobility and balance deficiency, predict the risk of falling, and distinguish the moderate-to-severe state of health [24, 66]. We believe that an improved mobility characterization could be achieved through daily in-home evaluation as subjects walk at their usual pace without expending too much effort to walk at their best, and problems could be identified earlier.

Several technological systems have been previously proposed, each with its own advantages and disadvantages. The majority of systems are either based on installed cameras or wearable sensors [43, 67]. However, installing cameras at home may violate a person's privacy, and wearable sensors may be forgotten generally. Accordingly, we propose to embed a Doppler radar system in the backrest of a smart chair for the TUG automation, and segmentation and kinematic analysis of the human gait cycle. Our proposed system can be installed at home (as well as in clinics) in a nonintrusive friendly manner and inexpensively, and can operate in variable lighting conditions while protecting human privacy requirements.

The present work provides supporting evidence for our three key findings: (1) automation of the TUG test with a Doppler radar system and a multiresolution analysis approach, (2) automatic segmentation of the TUG phases based on steps recognition and DARC algorithm, and (3) extraction of several important spatiotemporal gait parameters.

First, we aimed to validate the use of an MDU radar system in our work. The signal processing analysis was based on the continuous wavelet transform (CWT) approach, providing a multiresolution analysis by mapping the radar output into the time-frequency plane. Human gait speed signals (i.e., torso speed signals) were extracted after CWT analysis and compared with those obtained from the Vicon reference system. The correlation coefficient results support the hypothesis that our radar system works efficiently in automating the TUG test.

The second objective of our study was to automatically segment TUG phases (i.e., transfer, walk, and turn) that are currently neither timed by clinicians nor measurable easily through conventional hand-timed methods. Based on CWT results, we proposed two segmentation approaches. The first approach aimed to detect the toe speed signal and identify the first and last steps in walking to segment “transfer” and “walk” phases. Based on a threshold detection technique and the K-means approach, we clustered the energy distribution values into noise and limb oscillations. The K-means approach was transformed from a nonsupervised machine learning into a semisupervised one. We obtained evidence that setting initial centroids reasonably enhances clustering results while correlating with toe speed signals obtained after our analysis and those obtained from the Vicon system (*ρ*=0.89). Additionally, our methods indicate a good transfer-walk segmentation with a correlation coefficient between obtained and true time indices equal to 0.95. On the other hand, the second approach aimed to detect the first and last indices of turning while performing the TUG test in order to segment “walk” and “turn” phases. Based on the extraction of the variance parameter from the CWT matrix and the application of the DARC algorithm, we were able to obtain consistent results. A high correlation coefficient explains this reaching between the obtained and the reference time indices.

Our study's third objective was to assess the spatiotemporal parameters of gait and balance. This assessment could complete the TUG mobility evaluation by providing supplementary qualitative information and complementary analysis. Based on a preprocessing analysis of radar data, the TUG segmentation, and the extraction of torso and toe speed signals, we were able to provide valid equations for the estimation of 14 gait parameters such as cadence, stride length, step length, and swing time. These parameters are not measured by clinicians while performing the TUG test; however, they help in differential and detailed diagnosis. Our results are consistent with the reference values, showing a percentage of error varying between 0 and 4.8.

Although we obtained reliable results, certain limitations in this study could be addressed in future work. One limitation is that the number of participants in our experimental protocol is low. Additionally, the participants are aged between 22 and 60 years; but they were demanded to mimic elderly people by performing the TUG test at a slow speed. Several gait and posture characteristics commonly change with aging such as the decrease in walking speed and step length, the increase in the double support time, and the increase in the time needed to get up from a chair or sit down. According to the Doppler equation, the output frequency signal will decrease when the target speed decreases. In our study, the Doppler radar system was connected to an electronic circuit in order to amplify its output and filter out noise. The lower cutoff frequency of the bandpass filter was relative to the minimum walking speed, and the higher frequency was approximately relative to the highest frequency shift that could be generated from the human limb oscillations. Our proposition was tested and validated based on our collected data. Although they were asked to perform TUG trials at a slow speed to mimic elderlies' performance, however, it is significant to validate or adapt the cutoff frequencies to ensure an accurate analysis of elderly people with or without mobility difficulty while performing the TUG test. Accordingly, it would be useful to extend our data acquisition in future work with a new experimental protocol combining a heterogeneous group of people of different ages, ranging from young participants to older people, in order to ensure the validity and reliability of our approaches and results regardless a person's age, speed, and the TUG test performance.

In addition to this, the interpretation of TUG results is built on the concept of comparing outputs with a certain reference scale. According to the founder, an elderly subject who accomplishes the test in a total time of 10 seconds or less is considered to have normal mobility and autonomy in daily life activities. However, this reference scale could be based on the “vital signs of walking” of a group of elderly people who performed the test under specific conditions. We believe that several factors can alter how a person walks, gets up from a chair, and makes a turn. For example, Balzac's Theory offered a scientific and erudite way to describe human gait and discuss factors influencing gait [68]. Balzac admitted that weight, height, personality, occupation, social standing, either race or weather, and other psychological factors could influence gait. Additionally, as declared in Holmes and Holmes study [69], the world is made up of different cultures, subsequently, aging experiences appear at different scales. Thus, we can admit that seniors growing up in some countries have a walking pattern they go through that may not be identical or similar to those of other elderlies ageing in other societies or countries. Accordingly, in our opinion, the percentage errors are acceptable for the purpose of the TUG; however, it seems valuable to deliberate the factors influencing gait and balance into the reference scale in future work. Involving such references could help attain accurate results.

On the other hand, our proposed methodology was proposed for “Discrete Signal Processing.” However, we are tempted to suggest further improvements to automatically detect the starting and ending point of a TUG performance in order to implement our system for real-time operation. For example, we suggest extracting further parameters from the radar signals that could refer to a get up from a chair and a sit-down (i.e., starting and ending point of the test), so data between both intervals could be transferred and processed for analysis (show Figure 8). Another suggestion is to use a pressure sensor set on the armchair and/or the chair's backrest.

## 7. Conclusion

The TUG test is a practical, reliable, valid, ranked first assessment tool for mobility evaluation in older adults. In general, the time taken to perform the overall phases of the test is measured to analyze mobility in clinics under the supervision of a healthcare professional, thus unfrequently. Accordingly, in this study, we aimed first to automate the TUG test using an ambient sensor to provide a continuous, easy-to-use, and acceptable mobility evaluation at home. Then, we proposed automatic segmentation of TUG phases and estimation of gait parameters to afford complementary information. Our approaches were based on a multiresolution analysis and a semisupervised machine learning, and our results were promising as they indicated high correlations with those obtained from a reference system.

## Figures and Tables

**Table 1:** Error rate of the estimation of gait parameters.

Derived gait parameters	Percentage error
Number of steps; equation (13)	0
TUG duration (sec); equation (22)	4.8%
Step time (sec); equations (14) and (15)	∼4%
Gait cycle duration (sec); equation (16)	4%
Swing time (sec); equation (17)	∼6%
Average walking speed walk#1 & walk#2 (cm/sec); equations (18) and (19)	∼3%
Cadence (steps/min); equation (20)	4%
Step length (cm); equations (23) and (24)	3%
Stride length (cm); equations (25) and (26)	∼4.5%

## Data Availability

The data used to support our findings were generated during the study through an experimental protocol developed by the corresponding author at the University of Technology of Troyes, France.
